# Prediction of treatment response in lupus nephritis using density of tubulointerstitial macrophage infiltration

**DOI:** 10.3389/fimmu.2024.1321507

**Published:** 2024-02-13

**Authors:** Jingjing Wang, Wenyuan Lou, Mengyue Zhu, Yuanmao Tu, Duqun Chen, Dandan Qiu, Feng Xu, Dandan Liang, Zhen Cheng, Haitao Zhang

**Affiliations:** ^1^ National Clinical Research Center for Kidney Diseases, Jinling Hospital, Affiliated Hospital of Medical School, Nanjing University, Nanjing, China; ^2^ Northern Jiangsu People’s Hospital, Yangzhou University, Yangzhou, China

**Keywords:** lupus nephritis, macrophage infiltration, treatment response, predictor, predictive models

## Abstract

**Background:**

Lupus nephritis (LN) is a common disease with diverse clinical and pathological manifestations. A major challenge in the management of LN is the inability to predict its treatment response at an early stage. The objective of this study was to determine whether the density of tubulointerstitial macrophage infiltration can be used to predict treatment response in LN and whether its addition to clinicopathological data at the time of biopsy would improve risk prediction.

**Methods:**

In this retrospective cohort study, 430 patients with LN in our hospital from January 2010 to December 2017 were included. We used immunohistochemistry to show macrophage and lymphocyte infiltration in their biopsy specimens, followed by quantification of the infiltration density. The outcome was the treatment response, defined as complete or partial remission at 12 months of immunosuppression.

**Results:**

The infiltration of CD68^+^ macrophages in the interstitium increased in patients with LN. High levels of CD68^+^ macrophage infiltration in the interstitium were associated with a low probability of treatment response in the adjusted analysis, and verse vice. The density of CD68^+^ macrophage infiltration in the interstitium alone predicted the response to immunosuppression (area under the curve [AUC], 0.70; 95% CI, 0.63 to 0.76). The addition of CD68^+^cells/interstitial field to the pathological and clinical data at biopsy in the prediction model resulted in an increased AUC of 0.78 (95% CI, 0.73 to 0.84).

**Conclusion:**

The density of tubulointerstitial macrophage infiltration is an independent predictor for treatment response in LN. Adding tubulointerstitial macrophage infiltration density to clinicopathological data at the time of biopsy significantly improves risk prediction of treatment response in LN patients.

## Introduction

Lupus nephritis (LN) is a common and important manifestation of systemic lupus erythematosus (SLE). About 60% of patients with SLE experience renal involvement, and 10% to 20% progress to end-stage kidney disease (ESKD) (1, 2). Chronic kidney disease (CKD) and ESKD caused by LN are the leading causes of mortality among patients with SLE. Although the precise pathogenesis of LN is not very clear at present, it is widely believed that immune and autoimmune activation plays an important role. Pathologically, LN is characterized by immune complex deposition and inflammation in glomeruli and the tubulointerstitium (3). Therefore, glucocorticoid steroids combined with other immunosuppressants are recommended to treat LN. With the rapid progress in the treatment of LN, several new immunosuppression strategies that target different pathways in the pathogenesis of LN have recently emerged (4). Despite these advancements, a subset of patients remains unresponsive to current therapeutic interventions.

It has been desired that a physician can predict the treatment response of patients with LN based on the biopsy. Unfortunately, there is a lack of good biomarkers that predict the response to treatment. Previous studies have shown that hypertension, serum creatinine (SCr), chronicity index score (CIs), and other indicators can serve as predictors of treatment response (5–8). However, the prognostic value of the conventional clinicopathological features remains low. Immune cells, including T lymphocytes, B lymphocytes, and macrophages, play roles in the pathogenesis of LN. The density of the immune cell infiltration, especially macrophages, correlates with the severity of renal histologic lesions and the level of SCr at biopsy (9, 10). Previous studies have demonstrated that an increased number of macrophages is associated with impaired kidney function. After activation, macrophages can secrete proinflammatory cytokines and activate other immune cells (9, 11). Additionally, macrophages exhibit compromised phagocytic activity and a diminished ability to eliminate apoptotic cells, resulting in the excessive generation of autoantibodies in LN patients.

Based on the above studies, macrophages have been suggested to be closely associated with LN. Therapeutic interventions targeting macrophage function in LN patients have been explored, and ongoing studies are investigating the potential benefits of macrophage depletion (12, 13). Nevertheless, it is not known whether the density of macrophage infiltration can serve as a predictor for treatment response in LN. Therefore, we aimed to determine whether the extent of macrophage infiltration in kidney can predict the response to immunosuppression in LN patients.

## Methods

### Patients

This retrospective cohort study was conducted with the approval of the Institutional Review Board of Nanjing Jinling Hospital (2021NZKY-021-01). A total of 1,297 patients with biopsy-proven LN in the hospital from January 2010 to December 2017 were screened for the cohort. Their renal biopsy samples were assessed following the classification criteria established by the International Society of Nephrology/Renal Pathology Society (ISN/RPS) (14). The patients who met at least four of the criteria for SLE in the 1997 revised American College of Rheumatology classification were selected for the cohort. Baseline and follow-up data of the LN patients were obtained from the database of the National Clinical Research Center of Kidney Diseases, Jinling Hospital.

**Figure 1:**
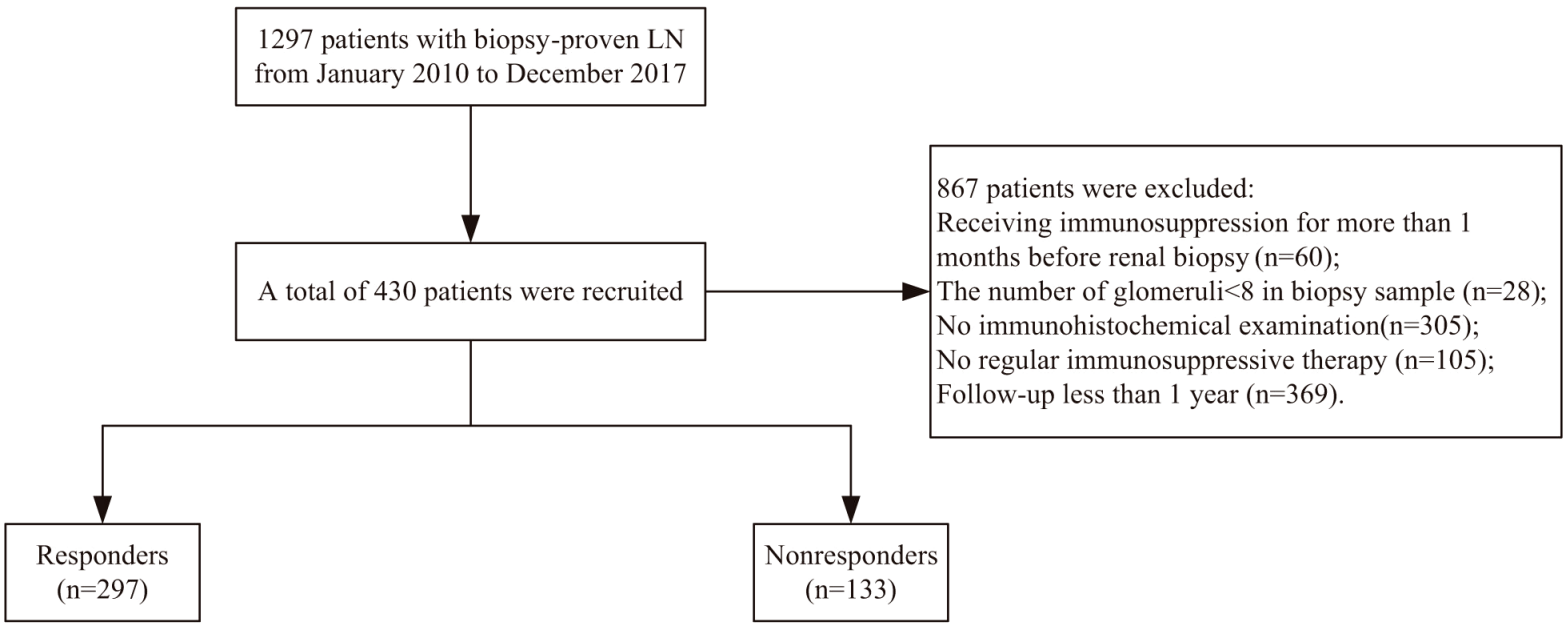
Flowchart of the study.

All the patients received regular immunosuppressive therapy for one year. Patients with incomplete information of CD4^+^, CD8^+^ lymphocytes, and CD68^+^ macrophages in kidney tissue and follow-up records were excluded (**Figure 1**). Ultimately, a total of 430 patients were enrolled in the study.

### Treatment response and definition

Baseline data collection and follow-up visits were conducted by research personnel. Data on demographic characteristics, clinicopathological features, treatment modalities, and treatment response were obtained. Anti-nuclear antibodies (ANA) and anti-double stranded DNA antibodies (anti-dsDNA) were described as positive or negative. The estimated glomerular filtration rate (eGFR) was calculated by the Chronic Kidney Disease Epidemiology Collaboration (CKD-EPI) (15). Disease activity was scored according to the Systemic Lupus Erythematous Disease Activity Index (SLEDAI). Treatment response included complete remission (urinary protein quantitation (UPRO) < 0.4 g/24 hours and normal SCr) and partial remission (≥ 50% reduction in proteinuria and UPRO < 3.5 g/24 hours, serum albumin (ALB) level ≥ 30 g/L, and normal or ≤ 25% increase in SCr level from baseline) at 12 months.

### Histopathology and macrophage infiltration in renal tissue

All renal biopsy specimens were obtained by percutaneous needle biopsy and were routinely examined by light microscopy, immunofluorescence, and electron microscopy. Two experienced pathologists independently examined the kidney biopsy specimens. They had access to the patient’s clinical information, including vital signs, blood test results, urinalysis results, and clinical diagnoses. Pathological parameters such as activity index score (AIs) and chronicity index score (CIs) were evaluated as described previously (16).

**Figure 2:**
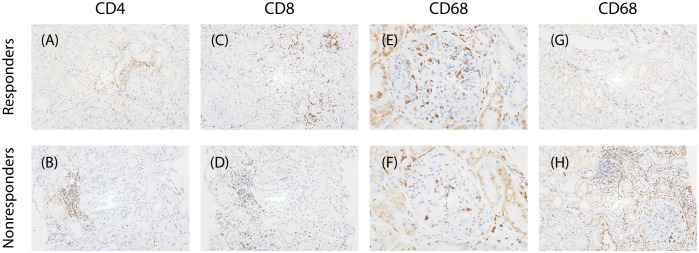
Characteristics of infiltrating cells in two groups. **(A, B)** Representative images of immunostaining for CD4 of tubulointerstitial (×200), **(C, D)** immunostaining for CD8 of tubulointerstitial(×200), **(E, F)** immunostaining for CD68 of glomerular (×400), **(G,H)** immunostaining for CD68 of tubulointerstitial(×200).

Infiltrating macrophages and lymphocytes in renal tissue were identified using immunohistochemical staining. Paraffin-embedded tissue sections (2 µm) were incubated with the following primary antibodies: mouse anti-human CD68 mAb (M0876; DAKO, Glostrup, Denmark), rabbit antihuman CD3 antibody (SP7; LEICA), rabbit antihuman CD4 antibody (NCL-L-368; LEICA) and rabbit antihuman CD8 (NCL-L-295). The slides were then incubated with the secondary antibody and visualized using a LEICA System Kit and counterstained with hematoxylin. The stained sections were scanned with a Digital Pathology Slide Scanner (Leica, Wetzlar, Germany). Positive-stained cells were automatically counted in all nonglobally sclerotic glomeruli at 400 magnifications using Aperio eSlide Manager (Leica)(**Figure 2**).

### Statistical analyses

The density of glomerular and tubulointerstitial infiltration between the response and nonresponse groups was compared using the Mann–Whitney test and the Spearman correlation between the density of glomerular and tubulointerstitial infiltration and clinicopathologic variables was calculated. In addition, logistic regression analyses were used to examine the association between different levels of glomerular and tubulointerstitial infiltration and the treatment response, with and without adjustment for gender, age, UPRO, SCr, C4, LN duration, hypertension, repeat biopsy, AIs, CIs, and ISN/RPS classification. Then all patients included in this study were randomly categorized into a training cohort or validation cohort in a 3:1 ratio. Three prediction models were used: a univariable model with the level of infiltration in glomeruli or tubulointerstitium separately; a model with CD4^+^ cells/interstitial field, CD8^+^ cells/interstitial field, and CD68^+^ cells/interstitial field together; and a model using combinations of the level of infiltration in glomerulus or tubulointerstitium, other pathological and clinical data. We also used stepwise regression with forward selection and backward elimination to obtain the model that minimizes the Alkaike information criterion (AIC). Performance of three logistic regression models in predicting the treatment response in both the training set and the validation set was reported and the predictive accuracy of these models was assessed by discrimination as measured by AUC.

Data analysis was performed using RStudio version 2023.03.0, an integrated development environment for R version 4.2.3 (R Core Team, Vienna, Austria). The difference was considered statistically significant as a two-sided P-value < 0.05.

## Results

### Characteristics of patients

Included in this study were 430 biopsy-proven LN patients, with the female gender predominating (84.65%) and a mean age of 30.31 ± 11.14 years (**Table 1**). The median SCr level at presentation was 0.83 (0.63–1.31) mg/dl. The median UPRO was 3.10 (1.72-5.58) g/24h. The median C3 and C4 levels were 0.41 (0.10-1.28) g/L and 0.08 (0.04-0.12) g/L, respectively. Most patients in our study had proliferative LN, of which 209 patients (48.60%) had pure proliferative LN (n = 35 for class III and n = 174 for class IV), 167 patients (38.83%) mixed proliferative LN (n = 44 for class III + V and n = 123 for class IV + V), 2 (0.47%) lupus podocytopathy, 5 (1.16%) class II LN, and 47 (10.93%) membranous LN. At the time of biopsy, the median AIs and CIs were evaluated by modified NIH activity and chronicity index of 7.00 (4.00-9.00) and 2.00 (1.00-3.00), respectively. The induction therapy was based on the histological lesions shown by renal biopsy. All the patients received treatment with corticosteroids without contraindications. Additionally, 126 (29.30%) received multi-target therapy, of which 120 cases of mycophenolate mofetil (MMF) combined with tacrolimus and 6 MMF combined with cyclosporine. There were 112 (26.05%) cyclophosphamide (CYC), 74 (17.21%) MMF, 66 (15.35%) calcineurin inhibitors (including 60 tacrolimus and 6 cyclosporine), 16 (3.72%) Triptergium wilfordii (TW), 7 (1.63%) rituximab (RTX), 6 (1.40%) azathioprine (AZA), and 5 (1.16%) autologous hematopoietic stem cell transplantation (AHSCT), respectively. 18 (4.19%) patients were treated with corticosteroids alone (**Table 1**).

**Table 1:** Baseline clinical characteristics of patients with lupus nephritis.

	Overall n=430	Responders n=297	Nonresponders n=133	P-value
Female gender,n (%)	364 (84.65%)	268 (90.24%)	96 (72.18%)	<0.001
Age (years)	30.31 ± 11.14	30.67 ± 11.18	29.50 ± 11.06	0.315
SLE duration (months)	6.00 (1.00-40.50)	6.00 (1.00-36.00)	12.00 (1.00-48.00)	0.015
LN duration (months)	2.00 (0.67-23.50)	1.00 (0.67-12.00)	4.00 (1.00-36.00)	0.018
SLEDAI (2K)	14.37 ± 5.28	14.80 ± 5.37	13.43 ± 4.96	0.013
Co-morbidities				
Hypertension, n (%)	136 (31.63%)	77 (25.93%)	59 (44.36%)	<0.001
Diabetes, n (%)	40 (9.30%)	27 (9.09%)	13 (9.77%)	0.822
SS, n (%)	9 (2.09%)	5 (1.68%)	4 (3.01%)	0.468
Extrarenal organ-system involvement, n (%)				
Cutaneous involvement	223 (51.86%)	158 (53.20%)	65 (48.87%)	0.407
Serositis	58 (13.49%)	36 (12.12%)	22 (16.54%)	0.215
Arthritis	200 (46.51%)	135 (45.45%)	65 (48.87%)	0.511
Neurological disorder	18 (4.19%)	12 (4.04%)	6 (4.51%)	0.822
Haematologic disorder	320 (74.42%)	217 (73.06%)	103 (77.44%)	0.336
Cardiac involvement	11 (2.56%)	4 (1.35%)	7 (5.26%)	0.017
Gastrointestinal involvement	47 (10.93%)	29 (9.76%)	18 (13.53%)	0.247
Laboratory parameters				
WBC (*10^9)	5.60 (4.23-8.30)	5.60 (4.30-8.40)	5.70 (4.20-8.20)	0.839
PLT (*10^12)	183.15 ± 79.43	183.63 ± 76.09	182.09 ± 86.74	0.853
Hb (g/L)	101.91 ± 20.13	103.00 ± 18.85	99.50 ± 22.61	0.096
ALB(g/L)	28.60 ± 5.87	28.82 ± 5.84	28.10 ± 5.93	0.240
BUN(mg/dl)	23.15 (15.43-38.45)	21.90 (14.90-33.10)	28.85 (17.70-55.80)	<0.001
SCr (mg/dl)	0.83 (0.63-1.31)	0.76 (0.62-1.04)	1.20 (0.70-2.25)	<0.001
UA (μmol/L)	400.50 (320.00-504.50)	379.00 (307.00-481.00)	439.00 (348.00-563.00)	<0.001
eGFR(mL/min per 1.73 m2)	96.44 (56.47-121.76)	103.55 (73.42-122.25)	64.98 (32.15-115.16)	<0.001
UPRO (g/24h)	3.10 (1.72-5.58)	3.30 (1.72-5.40)	2.95 (1.74-6.25)	0.962
Positive ANA, n (%)	414 (96.28%)	291 (97.98%)	123 (92.48%)	0.005
Positive anti-dsDNA, n (%)	245 (56.98%)	178 (59.93%)	67 (50.38%)	0.064
C3(g/L)	0.41 (0.10-1.28)	0.40 (0.10-1.28)	0.44 (0.10-1.23)	0.037
C4(g/L)	0.08 (0.04-0.12)	0.08 (0.04-0.10)	0.10 (0.06-0.15)	<0.001
C1q(U/ml)	25.29 (10.36-56.90) (n=381)	26.08 (10.79-62.08) (n=263)	21.70 (7.85-50.06) (n=118)	0.193
CD3^+^ cell counts (/μL)	790.50 (548.50-1082.75)	801.00 (566.00-1088.00)	749.00 (529.00-1053.00)	0.365
CD4^+^ cell counts (/μL)	303.50 (214.00-459.75)	312.00 (223.00-472.00)	259.00 (192.00-417.00)	0.006
CD8^+^ cell counts (/μL)	404.50 (265.00-585.75)	395.00 (262.00-554.00)	421.00 (274.00-611.00)	0.537
CD20^+^ cell counts (/μL)	163.50 (81.75-279.00) (n=400)	181.50 (98.00-308.25) (n=278)	119.00 (71.25-239.00) (n=122)	<0.001
Treatment				0.436
Pred+MMF+CNIs	126 (29.30%)	90 (30.30%)	36 (27.07%)	
Pred+CYC	112 (26.05%)	71 (23.91%)	41 (30.83%)	
Pred+MMF	74 (17.21%)	54 (18.18%)	20 (15.04%)	
Pred+CNIs	66 (15.35%)	45 (15.15%)	21 (15.79%)	
Pred only	18 (4.19%)	10 (3.37%)	8 (6.02%)	
Pred+TW	16 (3.72%)	14 (4.71%)	2 (1.50%)	
Pred+RTX	7 (1.63%)	4 (1.35%)	3 (2.26%)	
Pred+AZA	6 (1.40%)	5 (1.68%)	1 (0.75%)	
Pred+AHSCT	5 (1.16%)	4 (1.35%)	1 (0.75%)	

SLE, systemic lupus erythematous; LN, lupus nephritis; SLEDAI, Systemic Lupus Erythematous Disease Activity; SS, Sjogren’s syndrome; WBC, white blood cell; PLT, blood platelet; Hb, hemoglobin; ALB, albumin; SCr, serum creatinine; UA, blood uric acid; eGFR, the estimated glomerular filtration rate; UPRO, urinary protein quantitation; C3, complement 3; C4, complement 4; ANA, anti-nuclear; anti-dsDNA, anti-double stranded DNA antibodies; Pred, prednisone; MMF, Mycophenolate Mofetil; CNIs, Calcineurin Inhibitors; CYC, Cyclophosphamide; TW, Triptergium wilfordii; RTX, Rituximab; AZA, Azathioprine; AHSCT, Autologous hematopoietic stem cell transplantation.

### Clinicopathologic features in patients with or without response to treatment

Our analysis encompassed four patients who underwent repeat renal biopsies. The characteristics of the patients at the time of kidney biopsy were stratified by response to immunosuppression and are presented in **Tables 1**, **2**.

**Table 2:** Renal pathological features in the patients of lupus nephritis.

	Overall N=430	Responders n=297	Nonresponders n=133	P-value
Repeat biopsy	4 (0.93%)	4 (3.01%)	0 (0.00%)	0.009
Glomerular number	30.34 ± 11.80	31.33 ± 12.01	28.14 ± 11.05	0.009
ISN/RPS classification, n (%)				0.952
LP	2 (0.47%)	2 (0.67%)	0 (0.00%)	
Class II	5 (1.16%)	3 (1.01%)	2 (1.50%)	
Class III	35 (8.14%)	26 (8.75%)	9 (6.77%)	
Class III+V	44 (10.23%)	30 (10.10%)	14 (10.53%)	
Class IV	174 (40.47%)	119 (40.07%)	55 (41.35%)	
Class IV+V	123 (28.60%)	85 (28.62%)	38 (28.57%)	
Class V	47 (10.93%)	32 (10.77%)	15 (11.28%)	
AIs	7.00 (4.00-9.00)	7.00 (4.00-9.00)	6.00 (4.00-10.00)	0.788
CIs	2.00 (1.00-3.00)	1.00 (0.00-3.00)	2.00 (1.00-4.00)	<0.001
CD4^+^cells/interstitial field	92.00 (48.00-168.00)	76.00 (40.00-140.00)	144.00 (72.00-208.00)	<0.001
CD8^+^cells/interstitial field	96.00 (48.00-167.00)	76.00 (40.00-144.00)	148.00 (72.00-204.00)	<0.001
CD68^+^cells/glomerulus	9.80 (3.70-21.30)	11.00 (4.30-22.40)	7.40 (3.00-17.00)	0.011
CD68^+^cells/interstitial field	332.00 (184.00-504.00)	276.00 (168.00-452.00)	468.00 (280.00-636.00)	<0.001

LP, Lupus podocytopathy; AIs, activity index score; CIs, activity index score.

There were 69.07% of patients (n = 297) in the response group and the rest patients (n = 133) in the nonresponse group. The proportion of women in the response group was significantly higher than in the nonresponse group (P < 0.01). Patients in the response group were more likely to have higher SLEDAI scores (P = 0.013). However, patients in the nonresponse group were more likely to have comorbid hypertension, a longer course of SLE and LN, and a higher frequency of cardiac involvement (P < 0.05). Regarding laboratory parameters, patients in the response group were more likely to have better renal function, fewer immunological indexes, and more numbers of lymphocyte subsets than the nonresponse group (P < 0.05). There were no significant differences in age, diabetes, SS, induction therapy, and baseline level of leukocyte counts, platelets (PLT), hemoglobin (Hb), ALB, UPRO, Anti-Complement 1q antibodies (anti-C1q), CD3^+^ cell counts (CD3), and CD8^+^ cell counts (CD8) between the two groups (P > 0.05).

Histologically, there was no significant difference in pathological classification between the two groups. The patients in the nonresponse group had higher CIs compared with those in the response group (P < 0.05). Most importantly, the level of inflammatory infiltration including CD4^+^, CD8^+^ T cells, and CD68^+^ macrophages in tubulointerstitium was significantly higher in the nonresponse group. However, infiltrated CD68^+^ macrophages in glomeruli were increased in the response group.

### Correlations between cellular infiltration and clinicopathological parameters

**Figure 3:**
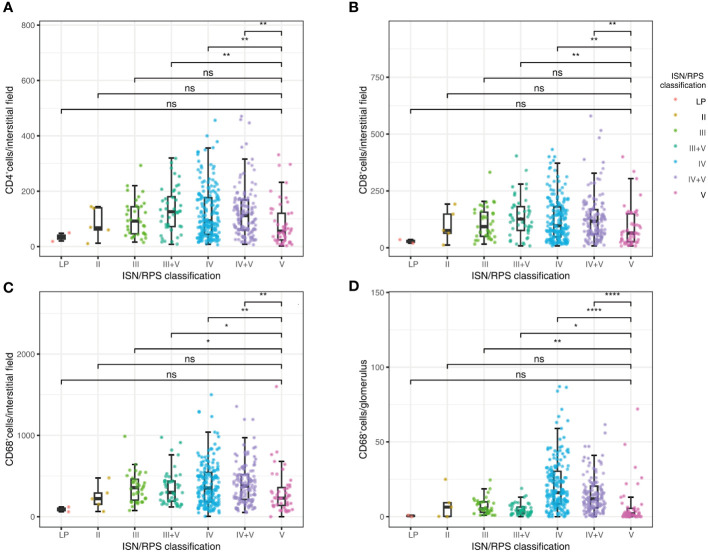
Correlations between ISN/RPS classification in LN with CD4^+^ lymphocyte infiltration in tubulointerstitium **(A)**, CD8^+^ lymphocyte infiltration in tubulointerstitium **(B)**, CD68^+^ macrophage infiltration in tubulointerstitium **(C)**, and CD68^+^ macrophage infiltration in glomeruli **(D)**. *P < 0.05, **P < 0.01, ***P < 0.001, ****P < 0.0001. Boxplot: boxplot medians (center lines), interquartile ranges (box ranges), whisker ranges; LP, lupus podocytopathy. ns, not significant.

We estimated the levels of CD4^+^, CD8^+^ lymphocytes, and CD68^+^ macrophages in glomeruli and tubulointerstitium in 430 patients with LN. As shown in **Figure 3**, **Table 3**, infiltrated CD4^+^, CD8^+^ lymphocytes, and CD68^+^ macrophages in tubulointerstitium were increased in patients with proliferative LN compared with those with pure SLE ISN/RPS class V. The number of CD68^+^ macrophages in glomeruli was also increased in patients with proliferative LN, but the statistical difference was not significant.

**Table 3:** The inflammatory cell infiltration in different ISN/RPS classifications of lupus nephritis.

	CD4^+^cells /interstitial field	CD8^+^cells /interstitial field	CD68^+^cells /glomerulus	CD68^+^cells /interstitial field
LP (n = 2)	34.00 (27.00-41.00)	28.00 (24.00-32.00)	0.50 (0.25-0.75)	90.00 (75.00-105.00)
Class II (n = 5)	68.00 (60.00-140.00)	76.00 (64.00-148.00)	6.40 (0.20-9.30)	220.00 (152.00-292.00)
Class III (n = 35)	92.00 (46.00-144.00)	92.00 (50.00-156.00)	5.30 (2.90-9.85)	356.00 (202.00-460.00)
Class III+V (n = 44)	126.00 (72.00-180.00)	126.00 (76.00-181.00)	3.70 (2.00-6.45)	298.00 (191.00-438.00)
Class IV (n = 174)	92.00 (44.00-176.00)	96.00 (48.00-180.00)	16.00 (8.67-30.27)	350.00 (184.00-544.00)
Class IV+V (n = 123)	112.00 (60.00-168.00)	116.00 (60.00-168.00)	11.70 (5.90-20.45)	376.00 (210.00-516.00)
Class V (n = 47)	56.00 (24.00-120.00)	64.00 (26.00-148.00)	2.00 (0.35-5.50)	228.00 (138.00-358.00)

LP, Lupus podocytopathy.

**Figure 4:**
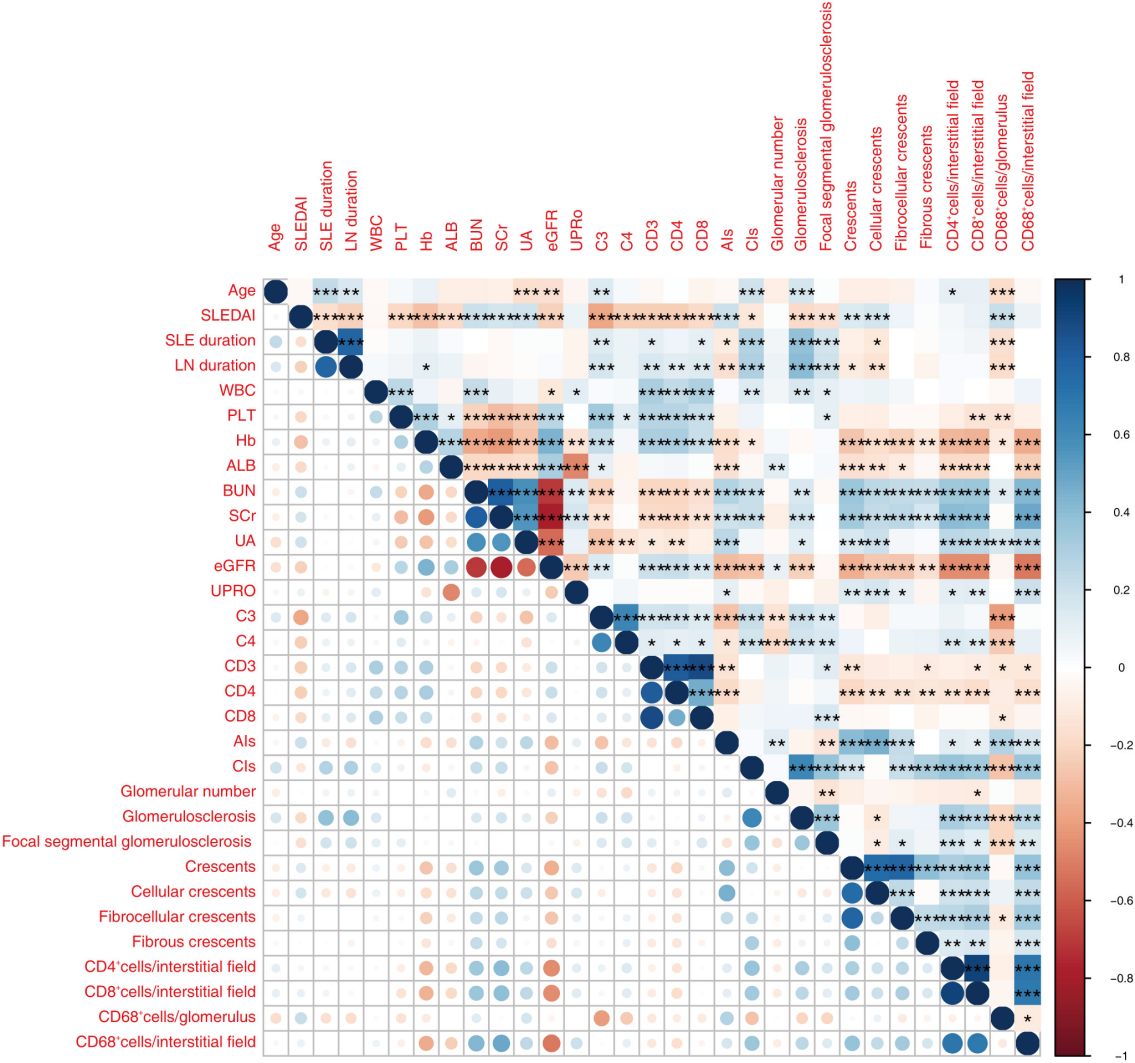
The heatmap shows correlations between parameters relevant for LN among the 430 patients enrolled. Only significant p values (P < 0.05) are shown. Red and blue colors represent significant negative and positive correlations. Darker color represents stronger correlations. *P < 0.05, **P < 0.01, ***P < 0.001.WBC, white blood cell; PLT, blood platelet; Hb, hemoglobin; ALB, albumin; SCr, serum creatinine; UA, blood uric acid; UPRO, urinary protein quantitation; eGFR, estimated glomerular filtration rate; C3, complement 3; C4, complement 4; CD4, CD4^+^ cell count; CD8, CD8^+^ cell count; AIs, activity index score; CIs, chronicity index score; GN, glomerulus number.

The density of infiltrated CD68^+^ macrophages in glomeruli correlated with age, SLEDAI, SLE duration, LN duration, PLT, Hb, blood urea nitrogen (BUN), UA, C3, C4, CD3, CD8, AIs, and CIs, with the highest Spearman correlation coefficients (r) being 0.41. The density of tubulointerstitial infiltration, including CD4^+^ cells, CD8^+^ cells, and CD68^+^ cells, positively correlated with BUN, SCr, and CIs, and negatively correlated with Hb and eGFR. The absolute values of r ranged from 0.31 to 0.51 (**Figure 4**, **Table 4**).

**Table 4:** The relationship between inflammatory cell infiltration and clinicopathological factors.

	CD4^+^cells/ interstitial field		CD8^+^cells/ interstitial field		CD68^+^cells/ glomerulus		CD68^+^cells/ interstitial field	
	r	P	r	P	r	P	r	P
Age	0.1028	0.0330	0.0913	0.0585	-0.1706	0.0004	-0.0324	0.5024
SLE duration	0.0391	0.4190	0.0535	0.2683	-0.1582	0.0010	-0.0096	0.8422
LN duration	0.0300	0.5354	0.0285	0.5560	-0.1724	0.0003	-0.0056	0.9083
SLEDAI (2K)	0.0445	0.3573	0.0835	0.0838	0.2164	<0.0001	0.0752	0.1192
WBC	0.0204	0.6727	-0.0061	0.8990	-0.0288	0.5510	0.0674	0.1628
PLT	-0.0877	0.0692	-0.1400	0.0036	-0.1457	0.0025	-0.0708	0.1429
Hb	-0.3120	<0.0001	-0.3313	<0.0001	-0.1053	0.0290	-0.3528	<0.0001
ALB	-0.1837	0.0001	-0.1871	0.0001	-0.0023	0.9626	-0.2322	<0.0001
BUN	0.3416	<0.0001	0.3535	<0.0001	0.1234	0.0104	0.4251	<0.0001
SCr	0.4015	<0.0001	0.3974	<0.0001	-0.0164	0.7350	0.4874	<0.0001
UA	0.2513	<0.0001	0.2405	<0.0001	0.1669	0.0005	0.2442	<0.0001
eGFR	-0.4514	<0.0001	-0.4543	<0.0001	-0.0486	0.3151	-0.5137	<0.0001
UPRO	0.1114	0.0209	0.1278	0.0080	-0.0067	0.8904	0.2048	<0.0001
C3	0.0763	0.1142	0.0376	0.4365	-0.4057	<0.0001	0.0128	0.7908
C4	0.1481	0.0021	0.1265	0.0087	-0.2462	<0.0001	0.0938	0.0520
CD3	-0.0764	0.1138	-0.0997	0.0389	-0.1137	0.0184	-0.1133	0.0187
CD4	-0.1367	0.0045	-0.1760	0.0002	-0.0943	0.0507	-0.1637	0.0007
CD8	-0.0151	0.7556	-0.0152	0.7530	-0.1078	0.0254	-0.0421	0.3842
AIs	0.1231	0.0106	0.1045	0.0303	0.2845	<0.0001	0.1615	0.0008
CIs	0.3688	<0.0001	0.3377	<0.0001	-0.2759	<0.0001	0.3552	<0.0001

SLE, systemic lupus erythematous; LN, lupus nephritis; SLEDAI, Systemic Lupus Erythematous Disease Activity; WBC, white blood cell; PLT, blood platelet; Hb, hemoglobin; ALB, albumin; SCr, serum creatinine; UA, blood uric acid; eGFR, estimated glomerular filtration rate; UPRO, urinary protein quantitation; C3, complement 3; C4, complement 4; CD4, CD4+ cell count; CD8, CD8+ cell count; AIs, activity index score; CIs, activity index score.

### Association between cell infiltration and response to treatment in patients with LN

The logistic regression analyses indicated that the tertile groups with more infiltration had a lower probability of responding to treatment in the crude model when they were compared with the groups with fewer CD4^+^, CD8^+^, and CD68^+^ cells infiltrated in the tubulointerstitium. The association between the density of CD68^+^ cell infiltration in the glomeruli and the treatment response disappeared even without adjustment (**Table 5**).

**Table 5:** Association between inflammatory cell infiltration and response to treatment in the study.

Exposure	Crude model	Model I	Model II
CD4^+^cells/interstitial field	0.994 (0.991, 0.996) <0.00001	0.996 (0.994, 0.999) 0.00727	0.997 (0.994, 1.000) 0.06717
Tertile 1 (2 – 56)	1.0	1.0	1.0
Tertile 2(60 - 136)	0.624 (0.350, 1.112) 0.10973	0.746 (0.407, 1.367) 0.34306	0.826 (0.429, 1.590) 0.56695
Tertile 3(140 - 472)	0.244 (0.140, 0.425) <0.00001	0.387 (0.208, 0.721) 0.00276	0.516 (0.260, 1.022) 0.05790
Trend	0.991 (0.988, 0.994) <0.00001	0.994 (0.990, 0.998) 0.00139	0.707 (0.503, 0.994) 0.04640
CD8^+^cells/interstitial field	0.995 (0.992, 0.997) <0.00001	0.997 (0.994, 0.999) 0.01472	0.998 (0.995, 1.000) 0.08402
Tertile 1(8 - 60)	1.0	1.0	1.0
Tertile 2(64 - 144)	0.713 (0.403, 1.262) 0.24540	0.873 (0.477, 1.597) 0.65879	0.952 (0.501, 1.809) 0.88034
Tertile 3(148 - 580)	0.236 (0.137, 0.406) <0.00001	0.381 (0.207, 0.703) 0.00201	0.514 (0.263, 1.004) 0.05139
Trend	0.991 (0.988, 0.994) <0.00001	0.994 (0.990, 0.997) 0.00065	0.700 (0.499, 0.982) 0.03891
CD68^+^cells/glomerulus	1.014 (0.999, 1.029) 0.07419	1.009 (0.992, 1.026) 0.31941	0.997 (0.979, 1.016) 0.76542
Tertile 1(0 - 5.5)	1.0	1.0	1.0
Tertile 2(5.6 - 15.8)	1.181 (0.723, 1.929) 0.50721	1.237 (0.714, 2.141) 0.44812	1.075 (0.577, 2.005) 0.81973
Tertile 3(16 - 87)	1.865 (1.120, 3.107) 0.01662	1.857 (1.007, 3.424) 0.04748	1.340 (0.645, 2.785) 0.43285
Trend	1.026 (1.005, 1.047) 0.01431	1.025 (1.000, 1.051) 0.04645	1.157 (0.804, 1.667) 0.43248
CD68^+^cells/interstitial field	0.997 (0.996, 0.998) <0.00001	0.998 (0.997, 0.999) 0.00041	0.998 (0.997, 0.999) 0.00522
Tertile 1(0.2 - 220)	1.0	1.0	1.0
Tertile 2(224 - 448)	0.637 (0.364, 1.115) 0.11439	0.814 (0.449, 1.477) 0.49837	0.817 (0.436, 1.529) 0.52679
Tertile 3(452 - 1600)	0.264 (0.155, 0.448) <0.00001	0.390 (0.213, 0.715) 0.00232	0.451 (0.231, 0.878) 0.01910
Trend	0.997 (0.996, 0.998) <0.00001	0.998 (0.996, 0.999) 0.00160	0.668 (0.477, 0.935) 0.01864

The crude model is not adjusted.

Adjust model I adjust for gender, age, UPRO, SCr, C4;

Adjust model II adjust for gender, age, UPRO, SCr, C4,LN duration, hypertension, repeat biopsy, AIs, CIs, and ISN/RPS classification.

After adjusting for gender, age, UPRO, UA, SCr, and C4, the density of interstitium infiltration, expressed as CD4^+^, CD8^+^, and CD68^+^ cells in the tubulointerstitium, remained significantly associated with treatment response, although this association was attenuated. The association between the density of CD4^+^ and CD8^+^ cells in the tubulointerstitium and treatment response disappeared after further adjusting for gender, age, UPRO, SCr, C4, LN duration, hypertension, repeat biopsy, AIs, CIs, and ISN/RPS classification. However, the density of CD68^+^ cells in the tubulointerstitium remained significantly associated with treatment response after further adjustment. Among different level groups, the density of CD68^+^ cells in the tubulointerstitium was significantly associated with treatment response in a high-level group (452–1600) (adjusted HR, 0.451; 95% CI, 0.231, 0.878) (**Table 5**).

### Predicting the response to treatment using the density of CD4^+^, CD8^+^ T cells, and CD68^+^ macrophages in tubulointerstitium

Among the univariable models, inflammatory cell infiltration in tubulointerstitium had good performance in predicting the response to treatment with AUCs of 0.6672, 0.6592, and 0.6973 for CD4^+^, CD8^+^, and CD68^+^ cells, respectively (**Table 6**).

**Table 6:** Performance of inflammatory cell infiltration, clinical data, or other renal pathological features for predicting response to treatment.

Variables	AUC (95% Confidence Interval)	
	Training Cohort (n=322)	Validation Cohort (n=108)
CD4^+^cells/interstitial field	0.6672(0.6004,0.7340)	0.6569(0.5479,0.7660)
CD8^+^cells/interstitial field	0.6592(0.5916,0.7268)	0.6491(0.5403,0.7579)
CD68^+^cells/interstitial field	0.6973(0.6324,0.7622)	0.6061(0.4882,0.7239)
CD4^+^cells/interstitial field, CD8^+^cells/interstitial field and CD68^+^cells/interstitial field	0.6975(0.6327,0.7623)	0.6288(0.5148,0.7427)
CD68^+^cells/interstitial field, other pathological and clinical data	0.7812 (0.7266,0.8358)	0.7315 (0.6010, 0.8620)

Multivariable models incorporating infiltrations of all the inflammatory cells in tubulointerstitium, including CD4^+^, CD8^+^, and CD68^+^ cells, did not improve the predictive performance as compared with the single density of CD68^+^ macrophages in tubulointerstitium. To select a model that is convenient for clinical use, we used stepwise regression to select the variables with a minimized Alkaike information criterion. The final model included eight variables, including sex, SLEDAI, SCr, ANA, C4, CD4, CIs, and the density of CD68^+^ macrophage in the tubulointerstitium. The model was logit (P)= -0.4844 + 0.6953 (if male) +0.0887*SLEDAI -0.5931*SCr -4.1868*C4 + 1.4451 (if ANA positive) +0.0010*CD4 -0.2040*CIs -0.0017*the density of CD68^+^ macrophages in tubulointerstitium, where p is the probability of being responsive to treatment. This model produced an AUC of 0.7812 (95% CI, 0.7266 to 0.8358) in the training cohort and 0.7315 (95% CI, 0.6010 to 0.8620) in the validation cohort, respectively (**Table 6**).

Compared with the model containing CD68^+^ macrophages only in tubulointerstitium, the final model significantly improved the risk reclassification, and net reclassification improved by 4.5%.

Therefore, our subsequent study was focused on the infiltration of CD68^+^ macrophages in the tubulointerstitium. The relationship between CD68^+^ macrophages and response to immunosuppression was further stratified by gender, age, SLEDAI, SCr, UA, eGFR, UPRO, anti-dsDNA, C3, C4, CD4, PLN (proliferative lupus nephritis), AIs, and CIs (**Table 7**).

**Table 7:** Relationship between the density of CD68^+^ macrophage in tubulointerstitium and response to treatment under different stratification.

Subgroupstratification		Cases	HR (95%CI)	P-value	P for interaction
Gender	male	66	0.998 (0.996, 1.000)	0.0194	0.7779
	female	364	0.997 (0.996, 0.998)	<0.0001	
Age	≤38	334	0.998 (0.997, 0.999)	<0.0001	0.0234
	>38	96	0.994 (0.991, 0.997)	0.0004	
SLEDAI	≤12	317	0.997 (0.996, 0.998)	<0.0001	0.8869
	>12	113	0.997 (0.995, 0.999)	0.0058	
SCr	≤1.2	312	0.999 (0.997, 1.000)	0.0470	0.4323
	>1.2	118	0.998 (0.996, 0.999)	0.0021	
UA	≤380	192	0.998 (0.996, 1.000)	0.0117	0.7326
	>380	238	0.997 (0.996, 0.999)	<0.0001	
eGFR	>60	118	0.997 (0.996, 0.999)	0.0005	0.1434
	≤60	312	0.999 (0.997, 1.000)	0.0831	
UPRO	≤2.5	184	0.997 (0.996, 0.999)	0.0004	0.8852
	>2.5	246	0.997 (0.996, 0.998)	<0.0001	
A-dsDNA	negative	185	0.997 (0.996, 0.999)	0.0001	0.8366
	positive	245	0.997 (0.996, 0.999)	<0.0001	
C3	≤0.46	254	0.997 (0.996, 0.998)	<0.0001	0.9764
	>0.46	176	0.997 (0.996, 0.999)	0.0001	
C4	≤0.09	248	0.998 (0.996, 0.999)	0.0002	0.7275
	>0.09	182	0.997 (0.996, 0.999)	<0.0001	
CD4	≤260	173	0.998 (0.996, 0.999)	0.0001	0.5375
	>260	257	0.997 (0.996, 0.998)	<0.0001	
PLN	NO	54	0.996 (0.993, 1.000)	0.0232	0.5639
	YES	376	0.997 (0.996, 0.998)	<0.0001	
AIs	≤3	66	0.997 (0.994, 1.000)	0.0311	0.8287
	>3	364	0.997 (0.996, 0.998)	<0.0001	
CIs	≤3	361	0.998 (0.997, 0.999)	<0.0001	0.4082
	>3	69	0.997 (0.995, 0.999)	0.0079	

SLEDAI, Systemic Lupus Erythematous Disease Activity; SCr, serum creatinine; UA, blood uric acid; eGFR, estimated glomerular filtration rate; UPRO, urinary protein quantitation; C3, complement 3; C4, complement 4; CD4, CD4+ cell count; LP, Lupus podocytopathy; AIs, activity index score; CIs, activity index score.

## Discussion

Our study has indicated that the density of CD68^+^ macrophage infiltration in the tubulointerstitium can predict the treatment response with high accuracy. Among the inflammatory cells tested, only the density of CD68^+^ macrophage infiltration in the tubulointerstitium at the time of renal biopsy was associated with response in the treatment for one year. The density of CD68^+^ macrophage infiltration was shown to be a more effective predictor of treatment outcome compared to conventional histological assessment. Furthermore, we have developed a simple prediction model that incorporates clinicopathological variables and CD68^+^ cell infiltration and has an AUC of 0.78.

In the majority of previous studies, several variables such as SCr, CIs, age, sex, Hb, urine protein-to-creatinine ratio (UPCR) or UPRO, LN duration, ISN/RPS classification, interstitial fibrosis, and tubular atrophy, have been identified as independent risk factors for poor treatment response in LN patients. In this study, eight factors were included in the prediction model, namely SCr, CIs, the density of CD68^+^ macrophages in the tubulointerstitium, sex, SLEDAI, ANA, C4, and CD4. Notably, the density of CD68^+^ macrophage infiltration in the tubulointerstitium demonstrated exceptional predictive ability for treatment response, even superior over the established factors such as SCr and CIs. Indeed, it has previously been suggested that urine macrophages could be a promising marker for identifying patients with active kidney disease in childhood-onset systemic lupus erythematosus (cSLE) (17). Also, one study showed that the number of CD68^+^ macrophages in the tubulointerstitium was an independent variable associated with poor renal outcomes in proliferative LN patients with a mean follow-up of 45 months (18).

Previous studies showed that macrophage infiltration density was closely associated with renal function decline in both transplanted kidneys and immunocomplex nephritis. Jan Hinrich Brasen et al. showed that the density of macrophages predicted future renal transplant function and improved the prognostic value of early renal transplant biopsies (19). Consistently, one study showed that tubulointerstitial macrophage infiltration was correlated with renal interstitial fibrosis and significant reduction of eGFR in patients with IgA Nephropathy (IgAN) (20), suggesting that renal tubulointerstitial macrophage infiltration was a potential hallmark of chronic kidney injury. Macrophages were shown to be necessary for the development of immune glomerulonephritis mediated by pathogenic antibodies in mouse models of LN, thus being a potential therapeutic target for LN (12, 21–23). In mouse models of lupus, functional impairment or depletion of macrophages resulted in attenuation of kidney injury (24, 25). Consistently, the reduction of macrophage recruitment to the kidney by disrupting the action of the related chemokines or their receptors also alleviated kidney injury in the lupus mice (26, 27). Similarly, macrophage injection or macrophage infiltration stimulation in lupus-prone mice aggravated nephritis and kidney damage (28).

At present, there is no panel of biomarkers or modeling approach for LN that exhibits superiority in predicting the response after treatment for one year. Quantification of tubulointerstitial macrophage infiltration is simple and robust, which may help improve the treatment decision for LN patients, particularly those who are at high risk of disease progression. After comparing the predictive performance of various types of inflammatory cell infiltration, we have developed a model combining tubulointerstitial macrophage infiltration and clinicohistological data in the present study. The results of response prediction to immunosuppression were consistent between training and validation cohorts. For simplicity and convenience, the final model uses only eight variables, nevertheless, it still shows good predictive performance. In addition, we conducted internal verification in the Pred+MMF+CNIs, Pred+CYC, Pred+MMF, and Pred+CNIs groups, and the AUC were 0.758, 0.719, 0.747, and 0.722 respectively, and there were no statistical differences between the groups.

This study has several limitations. Firstly, we excluded the patients whose data of CD4^+^, CD8^+^ lymphocytes, and CD68^+^ macrophages in the kidney biopsy sample and follow-up records were lacking, as noted earlier in the Methods. This exclusion might have resulted in both selection bias and confounding bias. Secondly, it was a pity that we did not have the quantitative data about the level of anti-dsDNA antibody, so we were unable to analyze the association between the level of anti-dsDNA antibody and response to treatment. Thirdly, the retrospective nature of this study prevented the collection of data on cumulative immunosuppressive agent dosage. Future prospective studies should be considered analyzing the correlation between cumulative immunosuppressive agent dosage and the level of CD68^+^ macrophage infiltration. Lastly, this is a single-center study conducted and validated in a Chinese population.

In conclusion, we have identified the density of macrophage infiltration in tubulointerstitium as an independent and robust predictor of response to immunosuppression in patients with LN. Our prediction model that combines the density of CD68^+^ macrophage in tubulointerstitium with clinicopathologic data can improve early treatment decisions for patients with LN.

## Data availability statement

The raw data supporting the conclusions of this article will be made available by the authors, without undue reservation.

## Ethics statement

The studies involving humans were approved by Institutional Review Board of Nanjing Jinling Hospital. The studies were conducted in accordance with the local legislation and institutional requirements. Written informed consent for participation was not required from the participants or the participants’ legal guardians/next of kin in accordance with the national legislation and institutional requirements.

## Author contributions

JW: Data curation, Investigation, Methodology, Writing – original draft, Writing – review & editing, Project administration, Validation. WL: Data curation, Investigation, Writing – original draft. MZ: Data curation, Investigation, Writing – original draft. YT: Data curation, Investigation, Writing – original draft. DC: Data curation, Investigation, Writing – original draft. DQ: Data curation, Investigation, Writing – original draft. FX: Methodology, Writing – original draft. DL: Methodology, Writing – original draft. ZC: Conceptualization, Supervision, Writing – review & editing. HZ: Conceptualization, Supervision, Writing – review & editing.
